# Nicotine and Its Action upon the Teeth

**Published:** 1883-07

**Authors:** David Hepburn


ARTICLE III.

### ON NICOTINE AND ITS ACTION UPON THE TEETH.

BY DAVID HEPBURN.

[Read before the Odontological Society, of Great Britain.]

MR. PRESIDENT AND GENTLEMEN:—I will occupy your time but for a few minutes this evening, in laying before you a short communication on "Nicotine and its Effects on the Teeth." I have been led to do so by the often-repeated query of patients as to whether or not the practice of smoking is deleterious to the organs of mastication, and I hope my few remarks may elicit your experiences upon this by no means unimportant question.

Notwithstanding the sweeping condemnation of the use of tobacco given by King James I., in his "Counterplaste" in which he describes it as "a custom loathsome to the eye, hateful to the nose, harmful to the brain, dangerous to the lungs, and in the black, stinking fume thereof nearest resembling the horrible Stygian smoke of the pit that is bottomless," the practice of smoking has increased up to the present day, and we find it employed as a universal luxury alike among civilized and savage nations. Snuff-taking has of late years decreased, chewing and smoking being more frequently indulged in. The former habit is, amongst our countrymen, chiefly confined to sailors; but in America I believe it is more universal. Although a distinct poison, the system, in the majority of instance as quoted by Dr. Arnott in an old volume of the *Lancet*, which show the enormous amount capable of being consumed, after prolonged practice, without creating apparent constitutional disturbance. The case was that of a sailor, aged 64, in the almost uninterrupted enjoyment of good health, who had chewed tobacco for upwards of fifty-years, also eating it. For many years in the latter part of his life he was in the constant practice of "eating a quarter of a pound of the strongest negro head every five days."

Taken internally, or inhaled in the form of smoke, the action of tobacco is that of a sedative and depressant; and in the unaccustomed, nausea is frequently produced. As a rule, however, a few trials will render the system tolerant of the poison, and when used in moderation it promotes regular action of the bowels, and soothes nervous irritation.

From a medical point of view it is interesting from the fact that it finds a place in the Pharmacopœia, but as a therapeutical agent it has been superseded by chloroform. In former times, however, an enema of tobacco was in rare cases prescribed, but great danger attend its administration. In this from it was capable of producing great muscular relaxation when that condition was necessary, as, for instance, in cases of strangulated hernia. *Nicotiana tabacum*, or Virginian tobacco, is class in company with many of our most deadly poisons—hyoscyamus, belladonna, and stramonium, in the natural order Solanaceæ; and it must not be confounded with Indian tobacco, or *Lobelia inflata*, of which there are two officinal preparations in the Pharmacopœia (*Tinctura labeliæ* and *Tinctura lobeliæ aetherea*), although they have much in common in their action upon the system.

The deadly nature of the alkaloid contained in tobacco may be seen from a quotation of the two following cases, in which death resulted from the administration of the drug. The first occurred in Belgium in 1851. An individual, it appears studied chemistry with a view to the preparation of the alkaloid nicotine. He administered the poison to his brother-in-law, and the victim succumbed in five minutes. The other case occurred in London in 1858: in this act was suicidal, and death was quite as rapid. It is said that the unfortunate individual, who was a chemist of rising reputation, was observed to stare wildly, and heaving a deep sigh died without convulsion. In addition, fatal results have followed its employment as an enema, and symptoms of an alarming nature have followed the inhalation of smoke, sleeping surrounded by bales of the weed, and even from carrying it next the skin, a practice which has been resorted to by smugglers.

From all parts of the tobacco plant, a liquid volatile alkaloid, *nicotina*, can be obtained, this nicotine when pure occurs as a colorless volatile oil, but on exposure to the air it becomes yellow, and this tint deepens by keeping. The taste is acrid, and the odor is discribed as "pleasant and ethereal." In this alkaloid is found the source of activity of the tobacco plant.

Nicotine is soluble in water, alcohol, and ether, and neutralizes acids. The analysis of tobacco smoke is of importance to us in considering this question. It is said to contain a certain amount of watery vapor. Ammonia is also present, and it is this which gives to the smoke an alkaline reaction. Next we have carbonic acid, to which Dr. Richardson has attributed much of the sleepiness and lassitude which follows prolonged inhalation of tobacco fumes. Further, it is stated that a certain amount of free carbon is always present. Lastly, we find the oil of tobacco which contains nicotine (the alkaloid), a volatile substance possessing a characteristic odor, and a dark resinous extract having a peculiar bitter taste. The quantity of this oil which is taken in at each inhalation of smoke may be easily seen by the old experiment of quickly *exhaling* a puff of smoke through a handkerchief stretched over the orifice of the mouth.

I have thus briefly enumerated some of the characteristics of this agent, which is so largely in use at the present day, and which, both in its secondary and direct effects, must act in a marked way upon the teeth of individuals who indulge in the practice of smoking. Of its secondary effects there is little to be said, save that in the fully matured, where the constitution has been impaired by the excessive use of tobacco, the result to the teeth will probably be similiar to that produced by any other influence tending to derange the bodily health—a subject too wide to enter upon on this occasion. Smoking, when indulged in before complete maturity, when the vitality of the teeth is peculiarly active in completing the structures of which they are composed, must exercise a most deleterious influence upon these organs and favor the development of decay. Consequently, what I may say of the direct action of nicotine on teeth, I intend only to apply where growth is complete.

From the slight observation I have given to this subject, I am led to believe that the direct action of nicotine upon the teeth is decidedly beneficial. The alkalinity of the smoke must necessarily neutralize any acid secretion which may be present in the oral cavity, and the antiseptic property of the nicotine tends to arrest any putrefactive changes which may be going on in carious cavities. But, in addition to these, we have another agent at work in the corbon with which tobacco smoke is impregnated, and I am inclined to believe that the dark deposit which we find on the teeth of some habitual smokers is largely composed of this ingredient.

It is this carbon which is deposited upon the back part of the throat and lining membrane of the bronchial tubes ; and with whatever disastrous effect it may act in these situations, from what we know of its wonderful antiseptic properties, I think we are justified in concluding that its action upon the teeth must be of a beneficial nature. Moreover, we find this deposit takes place exactly in those positions where caries is most likely to arise, and on those surfaces of the teeth which escape the ordinary cleansing action of the brush We find it interstitially, in all minute depressions, and filling the fissures on coronal surfaces. It may be removed with scaling instruments from the surface of the enamel but where it is deposited on dentine, this structure becomes impregnated and stained. Indeed, it is only where the enamel is faulty, and there is access to the dentine, that any true discoloration of the tooth takes place ; but it is remarkable how the stain will penetrate through even minute crocks, provided the necessary attention to cleanliness be not exercise. The staining power of tobacco oil may be seen when a deposit has taken place on the surfuce of tartar collected on the posterior surface of inferior incisors. In this situation a shiny ebony appearance is occasionaly produced That tobacco is capable of allaying, to some extent, the pain of toothache is, I think, true; its effect being due, not only to its narcotizing power, but also to direct action upon the exposed nerve; and I am inclined to attribute the fact of the comparatively rare occurrence of toothache amongst sailors, in great measure, to their habit of chewing. I have been struck, in the case of one or two confirmed smokers of an exaggerated type who have come under my notice, by the apparent tendency which exists towards the gradual production of complete necrosis of carious teeth, and the various stages of death of the pulp, and death of the periosteum taking place without pain or discomfort to the patient. This condition may, of course, be brought about by a variety of influences; but in these special cases I am inclined to think that the presence of nicotine in the mouth has acted powerfully. Hoping to hear your experiences on this subject of nicotine, and its effects on the teeth, I leave it in your hands.—*Dental Register.*

